# Chemogenetic modulation of histaminergic neurons in the tuberomamillary nucleus alters territorial aggression and wakefulness

**DOI:** 10.1038/s41598-021-95497-3

**Published:** 2021-09-09

**Authors:** Fumito Naganuma, Tadaho Nakamura, Hiroshi Kuroyanagi, Masato Tanaka, Takeo Yoshikawa, Kazuhiko Yanai, Nobuyuki Okamura

**Affiliations:** 1 Division of Pharmacology, Faculty of Medicine, Tohoku Medical and Pharmaceutical University, 1-15-1 Fukumuro, Miyagino-ku, Sendai, Miyagi 983-8536 Japan; 2 Department of Pharmacology, Tohoku University Graduate School of Medicine, 2-1 Seiryo, Aoba-ku, Sendai, Miyagi 980-8575 Japan

**Keywords:** Wakefulness, Non-REM sleep, Aggression

## Abstract

Designer receptor activated by designer drugs (DREADDs) techniques are widely used to modulate the activities of specific neuronal populations during behavioural tasks. However, DREADDs-induced modulation of histaminergic neurons in the tuberomamillary nucleus (HA^TMN^ neurons) has produced inconsistent effects on the sleep–wake cycle, possibly due to the use of Hdc-Cre mice driving Cre recombinase and DREADDs activity outside the targeted region. Moreover, previous DREADDs studies have not examined locomotor activity and aggressive behaviours, which are also regulated by brain histamine levels. In the present study, we investigated the effects of HA^TMN^ activation and inhibition on the locomotor activity, aggressive behaviours and sleep–wake cycle of Hdc-Cre mice with minimal non-target expression of Cre-recombinase. Chemoactivation of HA^TMN^ moderately enhanced locomotor activity in a novel open field. Activation of HA^TMN^ neurons significantly enhanced aggressive behaviour in the resident–intruder test. Wakefulness was increased and non-rapid eye movement (NREM) sleep decreased for an hour by HA^TMN^ chemoactivation. Conversely HA^TMN^ chemoinhibition decreased wakefulness and increased NREM sleep for 6 h. These changes in wakefulness induced by HA^TMN^ modulation were related to the maintenance of vigilance state. These results indicate the influences of HA^TMN^ neurons on exploratory activity, territorial aggression, and wake maintenance.

## Introduction

Histaminergic neurons in the tuberomamillary nucleus (TMN) of the posterior hypothalamus (HA^TMN^ neurons) modulate a multitude of physiological processes and behaviours, and altered neuronal histamine signalling is associated with changes in the sleep–wake cycle, motor activity and aggression among other behavioural effects^1,2^. Several technical challenges have limited research on the specific behavioural functions of HA^TMN^ neurons. Notably, histamine (HA) receptors are widely distributed throughout the central nervous system (CNS) and peripheral tissues, limiting the utility of pharmacologic modulation, while clusters of HA neurons may be difficult to stimulate specifically using implanted electrodes. Alternatively, chemogenetic and optogenetic techniques allow for the modulation of neuronal activities with regional, cellular and temporal specificity^3,4^. However, such techniques applied to HA^TMN^ neurons have also yielded inconsistent effects on the sleep–wake cycle. In one recent study, chemogenetic inhibition decreased wakefulness and increased non-rapid eye movement (NREM) sleep without changing REM sleep during active periods^5^. Similarly, another optogenetic study reported that acute silencing of HA^TMN^ neurons during wakefulness promoted NREM sleep, but not REM sleep^6^. However, Venner et al. reported that optogenetic inhibition of HA^TMN^ neurons during wakefulness did not alter the duration of NREM sleep. Furthermore, chemogenetic activation of HA^TMN^ neurons at Zeitgeber time (ZT) 3 did not alter the sleep/wake ratio or electroencephalogram (EEG) spectra^7^.

In addition to the sleep–wake cycle, the brain histaminergic system is involved in the regulation of locomotor activity and aggressive behaviours. Genetic deletion (knockout, KO) of histamine *N*-methyltransferase (*Hnmt*) (EC 2.1.1.8), which metabolises histamine into inactive 1-methylhistamine, induced a sustained elevation of extracellular histamine concentration in the mouse CNS and concomitantly reduced locomotor activity during the dark periods, enhanced aggressive behaviours and disrupted the normal sleep–wake cycle without changing the total amount of wakefulness^8^.

We speculated that these discrepancies in the behavioural effects of HA^TMN^ neuron modulation could reflect relatively minor differences in behavioural assessment methods and the emotional status of the animal. Furthermore, we suggest that the histidine decarboxylase (Hdc) (EC 4.1.1.22)-Cre mouse line may provide more cell type-specific chemogenetic or optogenetic manipulation of HA^TMN^ neuron activity^2,6^. Therefore, we re-examined histaminergic system functions in regulation of the sleep–wake cycle, locomotor activity and aggressive behaviours using designer receptor exclusively activated by designer drugs (DREADDs) in Hdc-Cre mice, and further examined the projection distribution of HA^TMN^ fibres by immunohistology to identify the neural circuits underlying the behavioural effects of HA^TMN^ neuron activity.

## Results

### Chemogenetic receptor expression by HA^TMN^ neurons after microinjection

**Figure 1:**
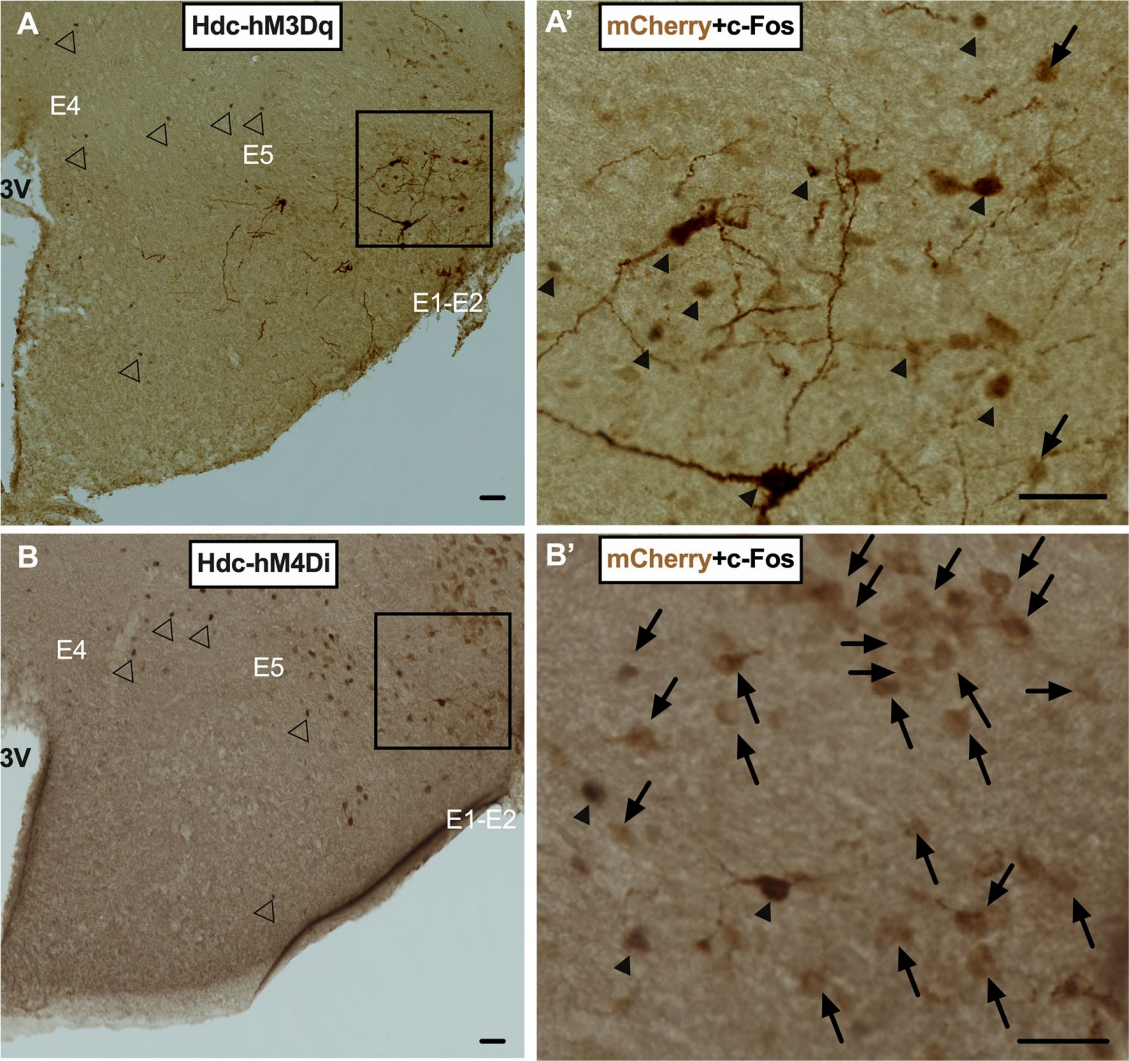
Specific expression of Hdc-hM3Dq/Hdc-hM4Di in the TMN and confirmation of neuronal activation or inhibition by CNO. (**A**) A representative hypothalamic section from an Hdc-Cre mouse injected with AAV-hM3Dq. CNO injection induced c-Fos expression (black nuclei) in mCherry-expressing HA^TMN^ neurons (brown) (**A′**). (**B**) Representative section from an Hdc-Cre mouse injected with AAV-hM4Di. c-Fos expression (black) was substantially lower in mCherry-positive neurons (brown) after CNO injection (**B′**). Scale bar = 200 μm. 3 V, third ventricle; E1-2 and E4-5, neuronal clusters of HA^TMN^. Arrows: mCherry-positive/c-Fos-negative neurons, Black arrow heads: mCherry-positive/c-Fos-positive neurons, White arrow heads: mCherry-negative/c-Fos-positive neurons. These coronal brain sections were located − 2.18 mm anterior–posterior from bregma.

Cell type-specific expression of chemogenetic receptors is crucial for establishing relationships between activity changes and behaviour. We therefore first confirmed that expression of chemogenetic receptors was exclusive to HA^TMN^ neurons in the posterior hypothalamus by scanning brain slices obtained after behavioural experiments for immunoreactivity to the vector marker mCherry. Indeed, mCherry immunoreactivity (brown) was detected only in posterior hypothalamus (TMN) in mice injected with hM3Dq (Fig. 1A) or hM4Di (Fig. 1B). Moreover, nuclear c-Fos expression (black) was markedly higher in hM3Dq-mCherry-immunoreactive cells (Fig. 1A′) compared to hM4Di-mCherry-immunoreactive cells (Fig. 1B′) 3.0 h after administration of clozapine N-oxide (CNO) (0.3 mg/kg i.p.), consistent with greater neuronal activity specifically in CNO responsive cells^9^.

### Activation of HA^TMN^ neurons enhanced locomotor activity in a novel environment

**Figure 2:**
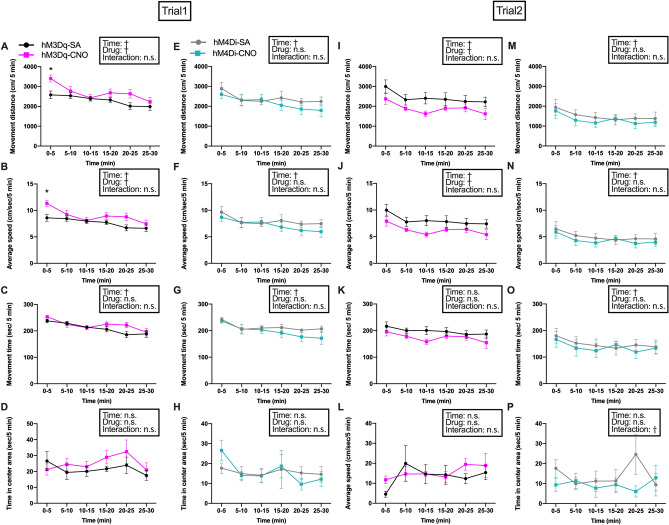
Enhanced locomotor activity in the open field (OF) by chemogenetic HA^TMN^ neuron activation depended on environmental novelty. Locomotor parameters were compared in the OF among Hdc-hM3Dq mice (n = 6, **A**‒**D**, **I**‒**L**) and Hdc-hM4Di mice (n = 5, **E**‒**H**, **M**‒**P**) for 30 min after CNO or SA injection by two-way RM ANOVA with main factors trial number (**A**‒**H**: novel OF in trial 1, **I**‒**P**: non-novel OF in trial 2) and drug treatment (CNO or SA), followed by Sidak's post hoc tests. (**A**) Total movement distance of Hdc-hM3Dq mice after CNO or SA injection during trial 1 (time: *F* = 7.48, *P* < 0.0001, drug: *F* = 5.77,* P* = 0.037, trial × drug interaction: *F* = 1.70, *P* = 0.15). (**B**) Average speed of Hdc-hM3Dq mice after CNO or SA injection during trial 1 (time: *F* = 7.48, *P* < 0.0001; drug: *F* = 5.77,* P* = 0.037; interaction: *F* = 1.70, *P* = 0.15). (**C**) Total movement time of Hdc-hM3Dq mice after CNO or SA injection during trial 1 (time: *F* = 8.77, *P* < 0.0001; drug: *F* = 2.91,* P* = 0.12; interaction: *F* = 1.46, *P* = 0.22). (**D**) Time spent in the central area by Hdc-hM3Dq mice after CNO or SA injection during trial 1 (time: *F* = 1.27, *P* = 0.29; drug: *F* = 1.02,* P* = 0.34; interaction: *F* = 0.77, *P* = 0.58). (**E**) Total movement distance of Hdc-hM4Di mice after CNO or SA injection during trial 1 (time: *F* = 9.92, *P* < 0.0001; drug: *F* = 0.46,* P* = 0.52; interaction: *F* = 1.80, *P* = 0.14). (**F**) Average speed of Hdc-hM4Di mice after CNO or SA injection during trial 1 (time: *F* = 9.96, *P* < 0.0001; drug: *F* = 0.46,* P* = 0.52; interaction: *F* = 1.80, *P* = 0.13). (**G**) Total movement time of Hdc-hM4Di mice after CNO or SA injection during trial 1 (time: *F* = 10.54, *P* < 0.0001; drug: *F* = 0.84,* P* = 0.39; interaction: *F* = 1.52, *P* = 0.21). (**H**) Time spent in the central area by Hdc-hM4Di mice after CNO or SA injection during trial 1 (time: *F* = 1.94, *P* = 0.11; drug: *F* = 0.0017,* P* = 0.97; interaction: *F* = 0.87, *P* = 0.51). (**I**) Total movement distance of Hdc-hM3Dq mice after CNO or SA injection during trial 2 (time: *F* = 3.44, *P* = 0.0096; drug: *F* = 5.05,* P* = 0.048; interaction: *F* = 0.31, *P* = 0.90). (**J**) Average speed of Hdc-hM3Dq mice after CNO or SA injection during trial 2 (time: *F* = 3.44, *P* = 0.0096; drug: *F* = 5.05,* P* = 0.048; interaction *F* = 0.31, *P* = 0.90). (**K**) Total movement time of Hdc-hM3Dq mice after CNO or SA injection during trial 2 (time: *F* = 2.05, *P* = 0.88; drug: *F* = 3.43,* P* = 0.094; interaction: *F* = 0.51, *P* = 0.77). **L,** Time spent in the centre area by Hdc-hM3Dq mice after CNO or SA injection during trial 2 (time: *F* = 2.22, *P* = 0.070; drug: *F* = 0.20,* P* = 0.67; interaction: *F* = 1.15, *P* = 0.35). (**M**) Total movement distance of Hdc-hM4Di mice after CNO or SA injection during trial 2 (time: *F* = 7.38, *P* < 0.0001; drug: *F* = 0.26,* P* = 0.62; interaction: *F* = 0.63, *P* = 0.68). (**N**) Average speed of Hdc-hM4Di mice after CNO or SA injection during trial 2 (time: *F* = 7.38, *P* < 0.0001, drug: *F* = 0.26,* P* = 0.62, interaction: *F* = 0.63, *P* = 0.68). (**O**) Total movement time of Hdc-hM4Di mice after CNO or SA injection during trial 2 (time: *F* = 5.98, *P* = 0.0003; drug: *F* = 0.12,* P* = 0.74; interaction: *F* = 1.27, *P* = 0.30). (**P**) Time spent in the centre area by Hdc-hM4Di mice after CNO or SA injection during trial 2 (time: *F* = 1.06, *P* = 0.40; drug: *F* = 0.54,* P* = 0.49; interaction: *F* = 3.45, *P* = 0.011). Data presented as mean ± S.E. ^†^*P* < 0.05 by two-way RM ANOVA for ‘time’ and ‘drug’, *P < 0.05 by post hoc Sidak’s multiple comparisons test.

Previous studies have yielded inconsistent or equivocal results regarding the effects of HA^TMN^ neuron excitation or inhibition on mouse locomotor activity. Acute pharmacological inhibition of histamine decarboxylase (HDC) or deletion of the *Hdc* gene, interventions that would reduce brain histamine levels, have been reported to decrease locomotor activity in rodents^10–14^. However, a chronic increase in histamine level also decreased locomotor activity in dark periods, while an acute moderate increase in brain histamine was found to enhance locomotor activity^8,15,16^. Histamine receptor stimulation/inhibition outside the TMN, effects of histamine on other neuronal populations and (or) compensatory changes after knockout may obscure effects of HA^TMN^ neurons on locomotion. Therefore, we established a DREADDs system to assess the effects of direct acute activation or inhibition of HA^TMN^ neurons. The chemogenetic activation of HA^TMN^ neurons with CNO did not significantly alter locomotor activity during the first 30-min trial compared with saline (SA) (Fig. S1A‒D). Similarly, inhibition of HA^TMN^ neurons by hM4Di-CNO did not decrease the mean locomotor activity during two 30-min trials compared with controls (Fig. S1E‒H). In the subgroup analyses, the hM3Dq-CNO group demonstrated increased total movement distance and average speed in the first trial (Fig. 2A,B). Time-resolved analyses indicated that these indices were significantly increased in the first 5-min period (Fig. 2A,B). In the second trial, however, hM3Dq-CNO significantly decreased movement distance and average speed (Fig. 2I,J). On the other hand, subgroup analysis of hM4Di did not reveal any significant differences (Fig. 2E‒H,M‒P). These results indicate that HA^TMN^ neuron activation enhances locomotor activity in a novel environment but actually decreases activity in a non-novel environment, suggesting that HA signalling influences exploratory behaviour.

### Chemoactivation of HA^TMN^ neurons increases territorial aggression

**Figure 3:**
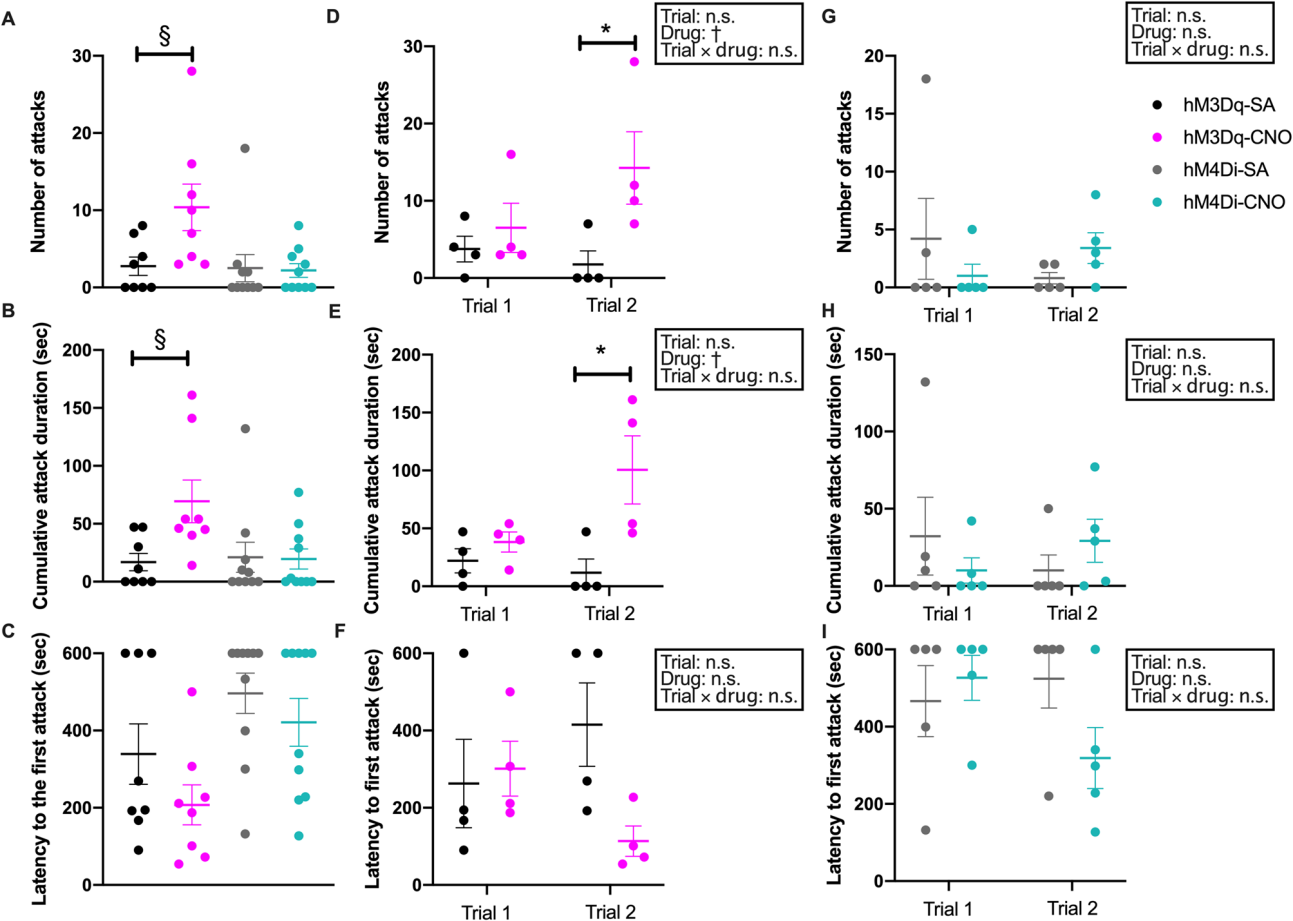
Chemogenetic activation of HA^TMN^ neurons enhanced aggression in the resident‒intruder test. The parameters of aggressive behaviours were compared b Hdc-hM3Dq mice (**A**, **B**, **D**, **E**, **G** and **H**) and Hdc-hM4Di mice (**A**, **C**, **D**, **F**, **G** and **I**) for 10 min after CNO or SA injection. (**A**, **D**, **G**) The combined results of trials 1 and 2, compared by Mann–Whitney U test (n = 8 or 10). (**B**, **E**, **H**) (n = 4), or (**C**, **F**, **I**) (n = 5) indicate results from each trial in Hdc-hM3Dq or Hdc-hM4Di mice, respectively. The results were compared by two-way RM ANOVA with main factors trial number (1 or 2) and drug treatment (CNO of SA), followed by Sidak’s post hoc tests. (**A**) Total number of aggressive behaviours (*P* = 0.027 by Mann–Whitney U test). (**B**) Total cumulative duration of aggressive behaviours (*P* = 0.013 by Mann–Whitney U test). (**C**) Total latency to first aggressive behaviour (no significant differences by Mann–Whitney U test). (**D**) Number of aggressive behaviours of Hdc-hM3Dq mice after CNO or SA injection in each trial (trial: *F* = 0.74, *P* = 0.42; drug: *F* = 7.51,* P* = 0.034; trial × drug interaction: *F* = 2.12, *P* = 0.20). (**E**) Cumulative duration of aggressive behaviours of Hdc-hM3Dq mice after CNO or SA injection in each trial (trial: *F* = 1.58, *P* = 0.26; drug: *F* = 16.68,* P* = 0.0065; interaction: *F* = 3.06, *P* = 0.13). (**F**) Latency to first aggressive behaviour of Hdc-hM3Dq mice after CNO or SA injection in each trial (n = 4; trial: *F* = 0.028, *P* = 0.87; drug: *F* = 3.89,* P* = 0.096; interaction: *F* = 2.58, *P* = 0.16). (**G**) Number of aggressive behaviours of Hdc-hM4Di mice after CNO or SA injection in each trial (trial: *F* = 0.059, *P* = 0.81; drug: *F* = 0.027,* P* = 0.87; interaction: *F* = 2.00, *P* = 0.12). (**H**) Cumulative duration of aggressive behaviours of Hdc-hM4Di mice after CNO or SA injection in each trial (trial: *F* = 0.0079, *P* = 0.93; drug: *F* = 0.011,* P* = 0.92; interaction: *F* = 1.50, *P* = 0.26). (**I**) Latency to first aggressive behaviour of Hdc-hM4Di mice after CNO or SA injection in each trial (n = 5; trial: *F* = 0.78, *P* = 0.40; drug: *F* = 1.12,* P* = 0.32; interaction: *F* = 2.44, *P* = 0.16). Data are presented as mean ± S.E.M. (horizontal bars and error bars) with superimposed individual data points (closed circles). ^†^*P* < 0.05 by two-way RM ANOVA with main factors ‘time’ and ‘drug’, *P < 0.05 by post hoc Sidak’s multiple comparisons test. ^§^*P* < 0.05. by Mann–Whitney U test.

Like locomotor activity, histaminergic influences on aggressive behaviours have been inconsistent across studies using pharmacologic manipulations or *Hnmt* KO^8,17–19^. Specific activation of HA^TMN^ neurons by hM3Dq-CNO significantly increased the number and duration of aggressive behaviours (Fig. 3A,B) compared to saline control in the resident–intruder test, and also tended to reduce the latency to first aggressive behaviour, although the difference did not reach statistical significance (Fig. 3C). The subgroup analyses of the inter- and intra-trials indicated that the specific activation of HA^TMN^ increased the number and duration of attack in the second trial (Fig. 3D,E). Conversely, inhibition of HA^TMN^ neurons by hM4Di-CNO had no significant influence on aggression compared to saline control (Fig. 3A–C,G–I). These results indicate that the activation of HA^TMN^ neurons triggers and maintains territorial aggression in mice.

### Modulation of HA^TMN^ neuron activity alters the sleep–wake cycle

**Figure 4:**
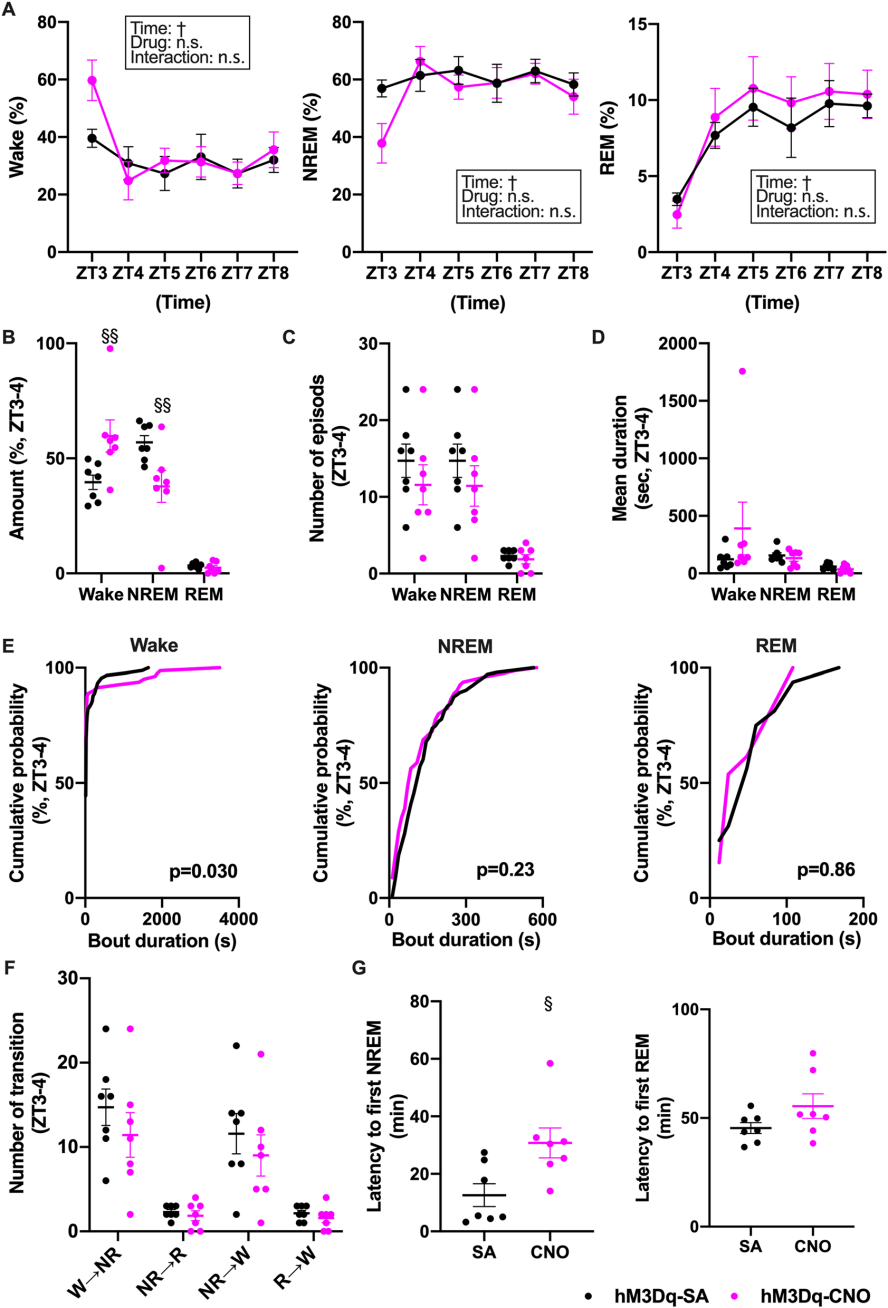
Chemogenetic activation of HA^TMN^ neurons increased wakefulness and decreased NREM sleep during light periods. Hdc-hM3Dq mice (n = **7**) were injected with CNO or SA at the indicated times and wake and sleep phases monitored by EEG/EMG. (**A**) Relative mean hourly wake, NREM sleep and REM sleep times (%) after CNO or SA injection at ZT3 (wake**-**time: *F* = 4.34, *P* = 0.0019; drug: *F* = 1.24, *P* = 0.29; time × drug interaction: *F* = 1.28, *P* = 0.29; NREM**-**time: *F* = 3.06, *P* = 0.016; drug: *F* = 1.31, *P* = 0.28; interaction: *F* = 1.46, *P* = 0.22; REM**-**time: *F* = 8.21, *P* < 0.0001; drug: *F* = 0.44, *P* = 0.52; interaction: *F* = 0.23, *P* = 0.95,; all by two-way RM ANOVA with main factors time and drug, followed by Sidak’s post hoc test). (**B**) Mean wake, NREM sleep and REM sleep times during ZT3-4 after CNO or SA injection at ZT3 (wake *P* = 0.0070 and NREM *P* = 0.0087 by Mann–Whitney U test). (**C**) Number of wake or sleep phase episodes during ZT3-4 after CNO or SA injection at ZT3 (no significant differences by Mann–Whitney U test). (**D**) Mean duration of sleep–wake state after CNO or SA injection at ZT3 (no significant differences by Mann–Whitney U test). (**E**) Vigilance state bout duration after CNO or SA injection at ZT3. Cumulative probability plots depict the relative distribution of the bout durations in each state during ZT3-4 (Wake *P* = 0.030, NREM *P* = 0.23 and REM *P* = 0.86 by Kolmogorov–Smirnov test). **F**, Frequencies of specific sleep–wake state transitions after CNO or SA injection at ZT3 (wake → NREM *P* = 0.30, NREM → REM *P* = 0.69, NREM → wake *P* = 0.36 and REM → wake *P* = 0.32 by Mann–Whitney U test). (**G**) Latency to the first NREM and REM sleep episode after CNO or SA injection at ZT3 (NREM *P* = 0.018 by Mann–Whitney U test). Data are presented as mean ± S.E.M. (horizontal bars and error bars) of n = 7 mice with (**B**–**D**, **F**, **G**) or without (**A**) superimposed individual data points (close circles). ^†^*P* < 0.05 by two-way RM ANOVA with main factors ‘time’ and ‘drug’; *P < 0.05 by post hoc Sidak’s multiple comparisons test; ^§^*P* < 0.05, ^§§^*P* < 0.01 by Mann–Whitney U test.

Next, we examined whether specific activation of HA^TMN^ alters the sleep–wake cycle by administering CNO or SA to AAV-hM3Dq mice at ZT3 (light period) or ZT12 (dark period). In two-arm crossover experiments, hM3Dq-CNO did not alter wake, NREM sleep, or REM sleep times within the first 6-h during light (Fig. 4A) or dark periods (Fig. S2A). Time-resolved analyses, however, revealed that CNO injection at ZT3 significantly increased total waking time and decreased NREM sleep time within the first 1 h (ZT3‒4) while REM sleep time was not changed (Fig. 4B). We also found that wake bouts were significantly skewed toward longer duration in the hM3Dq-CNO group by cumulative probability analyses (Fig. 4E left panel), while the number and mean duration of waking, NREM sleep and REM sleep episodes during the first 1 h were not changed (Fig. 4C,D, and Table S1). CNO administration to AAV-hM3Dq mice at ZT3 did not alter the frequencies of vigilance state transitions (Fig. 4F). On the other hand, the latency to first NREM sleep episode was significantly extended by hM3Dq-CNO (Fig. 4G). These results indicate that the activation of HA^TMN^ neurons contributes to arousal maintenance but not to vigilance state transitions. There were no differences in EEG power spectra during wakefulness, NREM sleep and REM sleep between CNO and SA groups (Fig. S4A). In addition, CNO injection at ZT12 did not alter sleep–wake state in sub-analyses (Figs. S2 and S4B, and Table S1).

**Figure 5:**
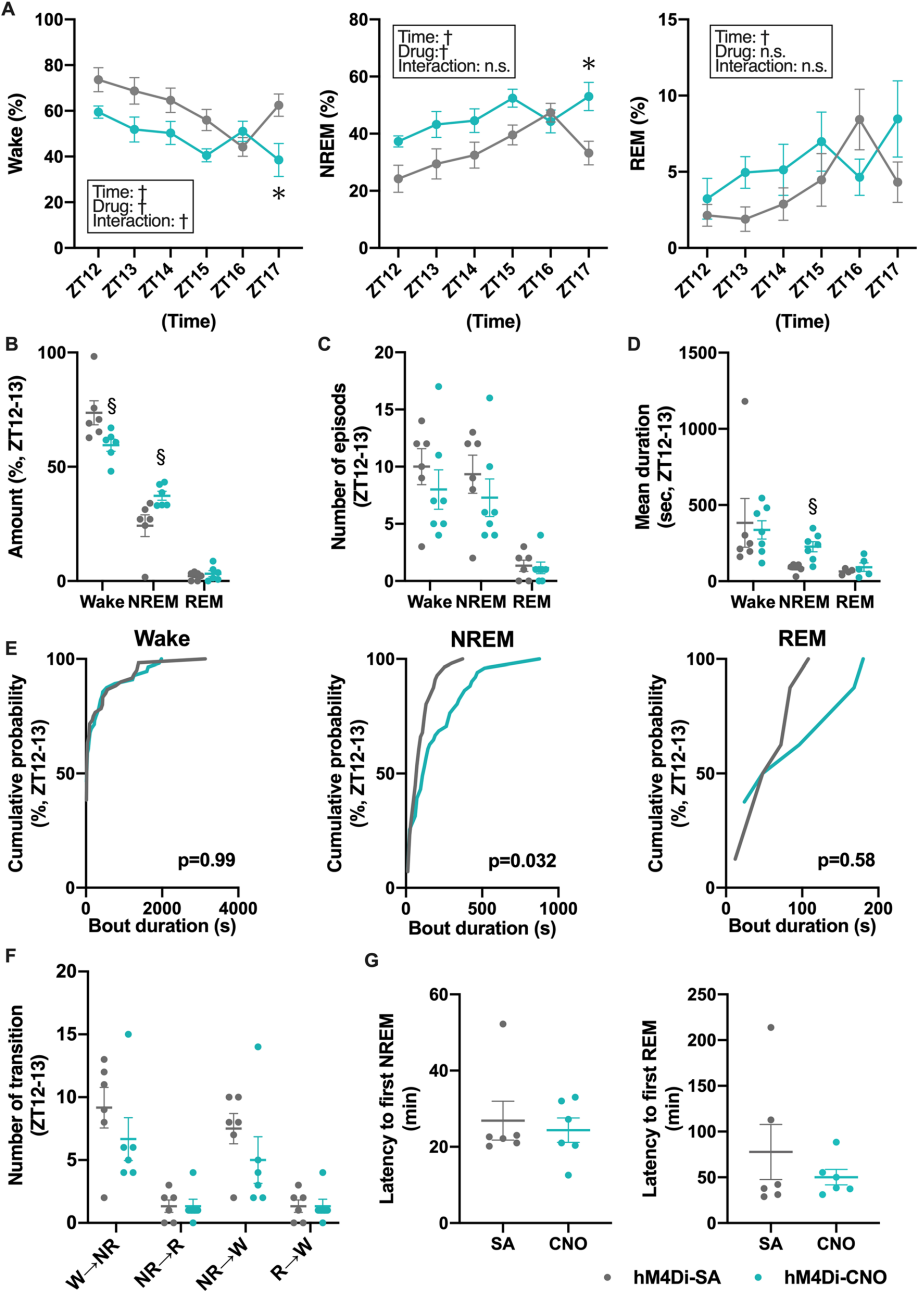
Chemogenetic inhibition of HA^TMN^ neurons decreased wakefulness and increased NREM sleep during dark periods. Hdc-hM4Di mice (n = 6) were injected with CNO or SA at the indicated time and wake and sleep phases monitored by EEG/EMG. (**A**) Hourly mean wake, NREM sleep and REM sleep durations after CNO or SA injection at ZT12 (wake**-**time: *F* = 5.99, *P* = 0.0002; drug: *F* = 10.02, *P* = 0.010; time × drug interaction: *F* = 2.81, *P* = 0.026; NREM**-**time: *F* = 5.65, *P* = 0.0003; drug: *F* = 10.39, *P* = 0.0091; interaction: *F* = 2.28, *P* = 0.061; REM**-**time: *F* = 3.18, *P* = 0.014; drug: *F* = 1.26, *P* = 0.29; interaction: *F* = 2.32, *P* = 0.057; all analyses by two-way RM ANOVA with main factors time and drug, followed by Sidak’s post hoc test). (**B**) Mean wake, NREM sleep and REM sleep durations during ZT12–13 after CNO or SA injection at ZT12 (wake *P* = 0.015 and NREM *P* = 0.013 by Mann–Whitney U test) (**C**) Number of episodes during ZT12-13 after CNO or SA injection at ZT12 (no significant differences by Mann–Whitney U test). (**D**) Mean duration of sleep–wake state after CNO or SA injection at ZT12 (no significant differences by Mann–Whitney U test). (**E**) Vigilance state bout duration after CNO or SA injection at ZT12. Cumulative probability plots depict the relative distribution of the bout durations in each state during ZT12-13 (Wake *P* = 0.99, NREM *P* = 0.032 and REM *P* = 0.58 by Kolmogorov–Smirnov test). (**F**) Sleep–wake state transitions after CNO or SA injection at ZT12 (wake → NREM *P* = 0.29, NREM → REM *P* = 0.93, NREM → wake *P* = 0.22 and REM → wake *P* = 0.93 by Mann–Whitney U test). (**G**) Latency to first NREM and REM sleep episodes after CNO or SA injection at ZT12 (no significant difference by Mann–Whitney U test). Data are presented as mean ± S.E.M. of n = 6 mice with (**B**–**D**, **F**, **G**) or without (**A**) superimposed individual data points (closed circles). ^†^*P* < 0.05 by two-way RM ANOVA with main factors ‘time’ and ‘drug’, *P < 0.05 by post hoc Sidak’s multiple comparisons test. ^§^*P* < 0.05 by Mann–Whitney U test.

We also examined whether specific inhibition of HA^TMN^ neurons suppressed wakefulness by comparing AAV-hM4Di mouse responses to CNO and SA administered at ZT3 or ZT12. Injection of CNO decreased waking time and increased NREM sleep time during the dark periods (Fig. 5A), while REM sleep in the dark periods and all sleep and wake stages in the light periods did not differ between CNO and SA groups (Figs. 5A and S3). Analysis of sleep–wake state during the first 1 h after CNO injection revealed decreased waking duration and increased NREM sleep duration but no changes in REM sleep duration (Fig. 5B). The mean duration of NREM sleep was significantly increased by hM4Di-CNO at ZT12 (Fig. 5D and Table S2), while the number of individual sleep or waking episodes, mean durations of wakefulness and REM sleep, frequencies of vigilance state transitions, and latencies to first NREM sleep and REM sleep episodes were not significantly changed (Fig. 5C,D,F,G). The distribution of the NREM bout durations was also significantly different between CNO and SA groups during ZT12-13 (Fig. 5E middle panel). Collectively, these findings demonstrate the HA^TMN^ neuron inhibition during the dark period impedes arousal maintenance. EEG power densities for each episode did not differ between CNO and SA groups (Fig. S4D). Further, CNO injection at ZT3 did not significantly alter sleep–wake state (Figs. S3 and S4C, Table S2). These results indicate that HA^TMN^ neuron activity during the dark period is crucial for maintaining arousal state.

### HA^TMN^ neurons projects to brain regions known to regulate aggression, vigilance and the sleep–wake cycle

**Figure 6:**
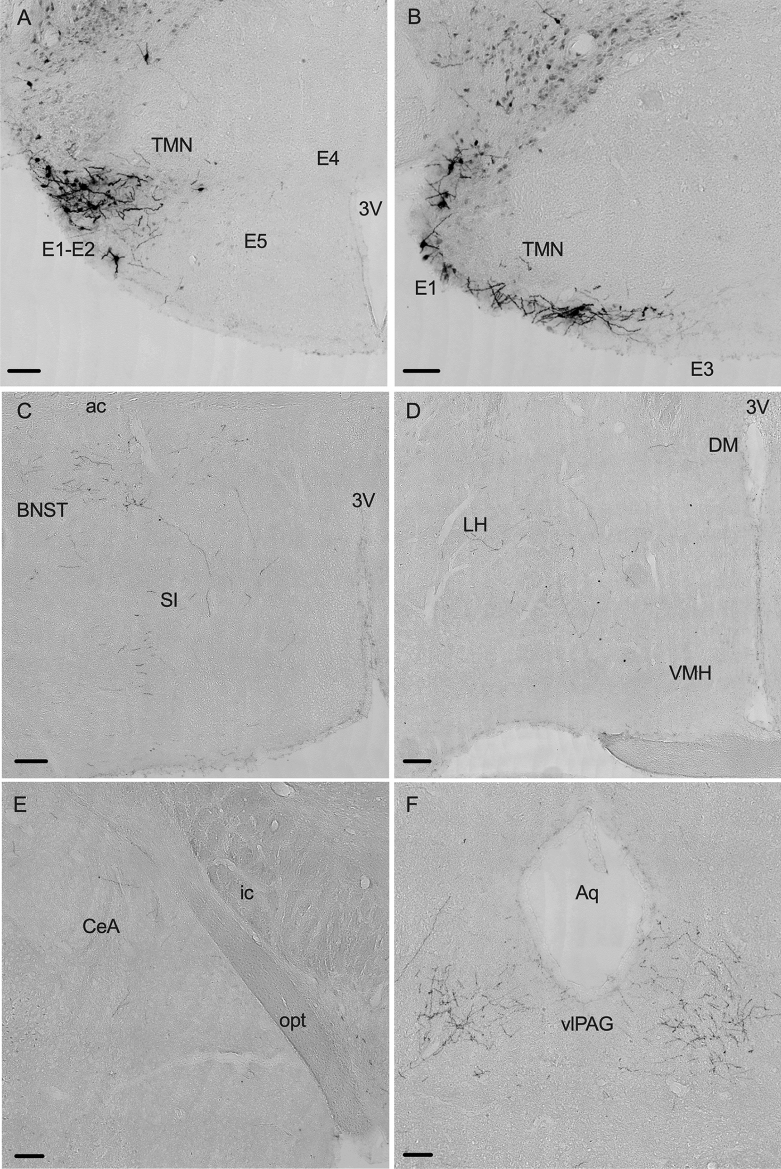
Projection targets of HA^TMN^ neurons. The presence of mCherry-immunoreactive HA^TMN^ somata and fibres were examined in coronal brain sections 6 months after AAV injections. (**A**) mCherry-immunoreactive HA^TMN^ neuronal somata and fibres in the ventrolateral TMN. (**B**) mCherry-immunoreactive HA^TMN^ neuronal somata and fibres in the caudal TMN. (**C**‒**F**) HA^TMN^ fibres in the bed nucleus of the stria terminalis (BNST) (**C**), lateral hypothalamus (LH) (**D**), central amygdala (CeA) (**E)** and ventrolateral periaqueductal grey (vlPAG) (**F).** Scale bar = 100 μm. *ac* anterior commissure, *Aq* aqueduct, *DM* dorsomedial hypothalamic nucleus, *LH* lateral hypothalamus, *opt* optic tract, *SI* substantia innominate, *TMN* tuberomamillary nucleus, *VMH* ventromedial hypothalamic nucleus, *3 V* third ventricle. E1–E5, neuronal clusters of HA^TMN^. Anterior–posterior locations (mm from bregma): (**A**) − 2.18 mm, (**B**) − 2.70 mm, (**C**) 0.14 mm, (**D**) − 0.46 mm, (**E**) − 1.34 mm and (**F**) − 4.24 mm.

We then investigated the potential circuits mediating these effects of HA^TMN^ neuron chemoactivation/chemoinhibition on locomotion, aggression and wakefulness by conducting serial mCherry immunostaining to identify projection targets. mCherry-immunoreactive somata were found exclusively in the ventral and caudal TMN 6 months after AAV microinjection (Fig. 6A,B). In contrast, mCherry-positive histaminergic fibres were observed in multiple brain regions at 6 months post-AAV microinjection, including the bed nucleus of the stria terminalis (BNST) and substantia innominata (SI) regions of the preoptic area (Fig. 6C), wide regions of hypothalamus but particularly in the lateral hypothalamus (LH) (Fig. 6D), central amygdala (CeA) (Fig. 6E) and periaqueductal grey (PAG), especially the ventrolateral PAG (vlPAG) (Fig. 6F). These regions may mediate the effects of HA^TMN^ neuron activity on exploratory locomotion, territorial aggression and the sleep–wake cycle.

## Discussion

In the present study, we demonstrate that the specific modulation of HA^TMN^ neuron activity by DREADDs can influence exploratory locomotion, territorial aggression and the sleep–wake cycle of mice. Although specific activation of HA^TMN^ neurons did not significantly alter overall locomotion in the two-arm crossover trial, activation moderately increased locomotion in trial 1 (first exposure) and moderately decreased locomotion in trial 2. In contrast to these modest effects on locomotion, activation of HA^TMN^ neurons markedly enhanced aggression of male mice toward an intruder male. Furthermore, HA^TMN^ neuron activation increased wakefulness for an hour in the light periods, whereas inhibition significantly decreased wakefulness during the dark periods. Transitions in sleep–wake state were also regulated by HA^TMN^ neuron activity. Finally, we show that TMN histaminergic neurons project to brain regions implicated in fear, aggression and arousal, such as the BNST, LH, CeA and vlPAG.

The HA^TMN^ neurons of rodents are distributed in five clusters, E1, E2 and E3 in the ventral area, E4 in the medial dorsal area and E5 more diffusely in the dorsal area^20^. At 2–3 months after injection of Hdc-hM3Dq or Hdc-hM4Di vector, mCherry-positive histaminergic neurons were localised primarily in lateral E1 and E2 (Fig. 1A,B), a distribution comparable to that of Hdc-Cre mice crossed with a Cre-dependent tdTomato reporter line^6^. In addition, however, immunostaining of brain sections 6 months after microinjection revealed additional mCherry-positive histaminergic neurons in E3 and E5 (Fig. 6A,B). A transgene downstream of the human synapsin gene (hSyn) promoter in the AAV vector was exclusively expressed in neurons, and AAV8 induced long-term upregulation of the transgene^21,22^. Therefore, a wider distribution of immunoreactive histaminergic neurons in the present study may result not only from a longer time period for expression but also from use of the hdc promoter. While we assume that the observed behavioural effects are due mainly to the activities of E1 and E2 histaminergic neurons (Fig. 1), contributions of E3 and E5 neurons cannot be totally excluded.

Previous studies have clearly demonstrated that HA^TMN^ neurons project to broad regions of the brain, including BSNT, LH, CeA and vlPAG^23,24^. Our projection study confirmed these findings and revealed a particularly dense projection to the bilateral vlPAG (Fig. 6F). Neurons of the vlPAG are involved in the regulation of locomotor activity^25^, aggressive behaviours^26^ and the sleep–wake cycle^27–29^, consistent with our behavioural results. Antegrade and retrograde optogenetics experiments are warranted to elucidate the precise circuit mechanisms.

A positive association between hypothalamic histamine release and physiological locomotor activity was first reported in 1992^30^ and thereafter evidence has accrued for important contributions of HA^TMN^ neuronal excitation to locomotor activity, mainly based on experiments in which histamine production or H_1_ and H_3_ receptors were inhibited^10,13,31,32^. In these loss-of-function studies, locomotor activity was generally reduced by inhibition of histaminergic signalling in the CNS, but gain-of-function studies in which brain histamine levels were increased have yielded unexpected and inconsistent results^8,15,16^. We found no significant difference in locomotion between CNO and control groups in the two-arm crossover experiment (Fig. S1), even in subgroup intracohort analysis between trials (data not shown). However, we found that HA^TMN^ activation increased locomotor activity in the Hdc-hM3Dq intercohort analysis for trial 1 (Fig. 2A,B) and decreased activity in trial 2, suggesting that HA^TMN^ neuron activation enhances locomotor activity in a novel environment^31,33^. Increased locomotor activity by specific HA chemogenetic activation was also reported by Yu et al. but they observed a greater and more sustained increase probably because of the higher dose of CNO used^34^. On the other hand, HA^TMN^ neuron activation decreased locomotor activity in trial 2 (Fig. 2I,J). Histaminergic activation also contributes to memory retrieval^35–37^, so this reduced locomotor activity in trial 2 may be due to strong contextual memory for an otherwise anodyne environment, resulting in lower exploratory motivation. In contrast, HA^TMN^ suppression by hM4Di-CNO did not significantly alter locomotor activity (Figs. 2 and S1). However, a previous study demonstrated that chemogenetic inhibition of HA^TMN^ by 1 mg/kg CNO injection significantly decreased locomotion in dark periods^5^. We performed open-field tests with a much lower CNO dose during the light periods, the resting phase for nocturnal animals, so further studies are needed to evaluate effects of loss-of-function on locomotor activity during the dark periods.

In contrast to the subtle effects on locomotion, HA^TMN^ neuron activation clearly enhanced territorial aggression (Fig. 3). Previous studies have suggested that elevated brain histamine levels induce aggression via H_2_ receptor activation^8,17,18^, consistent with the current results. Kárpáti et al., however, found that H_1_ receptors in astrocytes also play an important role in the suppression of aggressive behaviours^38^. Moreover, some HA^TMN^ neurons contain GABA as a cotransmitter^34^, which might be involved in aggression. These observations indicate that neurotransmitters released from several subtypes of HA neurons possibly modify aggressive behaviours via multiple receptors expressed on neurons and astrocytes. Thus, further studies are necessary to elucidate the molecular and cellular mechanisms of central histaminergic system in aggressive behaviours.

We speculate that two mechanisms underlie increased aggression upon HA^TMN^ neuron activation, direct modulation of neural pathways mediating aggression and modulation via effects on the circadian rhythm. One potential pathway is the ventromedial hypothalamus, ventrolateral subdivision (VMHvl)–vlPAG circuit^39^, as the VMHvl is innervated by the HA^TMN^ and induces aggression upon stimulation^24,40^. The HA^TMN^ also innervates the vlPAG (Fig. 6), which transforms higher level neuronal signals into aggressive action-specific codes^39^. A second possible pathway is the suprachiasmatic nucleus (SCN)–subparaventricular zone (SPZ)–VMHvl circuit. The SCN is the master circadian pacemaker in the mammalian brain, and sends dense axonal outputs to the SPZ, which negatively regulates aggression via the VMHvl^41^. The HA^TMN^ also projects to the SCN^24,42^ and has an inhibitory effect on SCN neuronal activity through H_1_ and H_2_ receptor activation^43–45^. Additional projection mapping and chemogenetic activation studies are required to clarify the contributions of these pathways to territorial aggression induced by HA^TMN^ neuron excitation.

To our knowledge, only three previous studies have used Hdc-Cre mice to examine the functions of HA^TMN^ neurons in regulation of the sleep–wake cycle^5–7^. Chemogenetic inhibition of HA^TMN^ neurons during the dark periods decreased wakefulness and increased NREM sleep duration in a different Hdc-Cre mouse line, and these responses were accompanied by increased EEG δ power and decreased EEG power at higher frequencies (8–30 Hz)^5^. A study using the same Hdc-Cre line employ in the current study found that optogenetic inhibition of HA^TMN^ neurons by archaerhodopsin (Arch) 3.0 during wakefulness in the dark periods promoted NREM sleep and increased the number of NREM episodes but not NREM duration, and had no effect on EEG spectra^6^. Another study demonstrated that zolpidem increased NREM sleep without significant changes in EEG power in the mice whose HA^TMN^ neurons were selectively modified to zolpidem-sensitive^46^. Although we found prolonged mean NREM duration without alteration in the number of episodes (Fig. 5C,D) and no differences in the EEG power spectra for wake and all sleep stages (Fig. S4), the inhibitory experiments in the present study revealed an important role for HA^TMN^ neurons in maintaining wakefulness during the dark periods, in accord with the aforementioned studies. However, inhibition of HA^TMN^ neurons with ArchT during wakefulness did not impact NREM sleep in experiments using another Hdc-Cre line^7^. These authors also induced chemogenic activation of HA^TMN^ neurons by CNO injection at ZT3 and found no alterations in the relative durations of sleep and wake stages or EEG spectra^7^. In the present study, we also found no changes in the sleep–wake cycle following HA^TMN^ neuron activation in the overall analysis (Fig. 4A–C). In sub-analysis, however, Hdc-hM3Dq mice demonstrated increased wakefulness in the first hour after CNO injection at ZT3 (Fig. 4B). The increased wakefulness was a consequence of the prolonged latency to first NREM and a higher probability of longer wake bouts by HA^TMN^ activation (Fig. 4E,G).

Previous electrophysiological studies have identified some TMN neurons as ‘Waking-on’ due to predominant activation during wakefulness and complete silence during NREM and REM sleep^47–49^. These neurons also exhibited a pronounced delay in firing during transitions from sleep to wakefulness and a long delay to an arousing stimulus, indicating important contributions to the maintenance of wakefulness and vigilance, but not to state induction^48^. Our findings from bout distributions and transition analyses, which showed prolonged wake bout duration by HA^TMN^ activation and prolonged NREM duration by HA^TMN^ inhibition provide further support for the role of HA^TMN^ neurons in arousal maintenance (Figs. 4E,F and 5E,F). Steininger et al. also suggested that some histaminergic neurons in rats discharged at high rates during REM sleep, termed ‘REM-related neurons’^47^. However, we found no relation between HA^TMN^ neuron activity and REM sleep.

As previously described, the Hdc-Cre transgenic mouse line used in the present study has high specificity with minimal Cre recombinase expression outside the TMN; however, it also exhibits only ~ 50% penetrance^6^. Thus, at least half of the Hdc-neuronal population was not modulated by the chemogenetic approach, which may explain why we did not observe the significant changes in locomotor activity and REM sleep reported in previous studies that used different Cre recombinase lines with higher penetrance^5,7^. These Hdc-Cre mouse lines, one a transgenic model^7^ and the other a knock-in model^34,46,50^, however, have higher ectopic expressions of Cre recombinase which were observed in various brain regions^2^. Further, several studies suggest that HA^TMN^ neurons are functionally heterogeneous^6,51–53^ and form distinct projections to influence unique physiological functions and behaviours. Further work is required to determine the individual HA^TMN^ pathways responsible for the observed changes in locomotor activity, aggression and the sleep–wake cycle. Furthermore, we found no correlations between behavioural changes across groups (data not shown). Larger cohorts for greater statistical power and projection-specific activation methods may be required to identify such relationships.

In conclusion, the specific modulation of HA^TMN^ neuron activity altered exploratory locomotion, territorial aggression and wake maintenance in mice. These responses may be explained by projections from HA^TMN^ neurons to the POA, CeA, LH and vlPAG.

## Materials and methods

### Animals

All animal care and use protocols were conducted in accordance with the Standards of Humane Care and Use of Laboratory Animals of Tohoku Medical and Pharmaceutical University, Sendai, Japan. All experiments involving animals also complied with the Guidelines for the Proper Conduct of Animal Experiments of the Ministry of Education, Culture, Sports, Science and Technology, Japan, and the ARRIVE guidelines for reporting animal research^54^. All animal experiments including the use of genetically modified mice were approved by the Tohoku Medical and Pharmaceutical University Animal Experiment Committee (registration number: 19004-cn) and Tohoku Medical and Pharmaceutical University Centre for Gene Research (registration number: 2018-19). All efforts were made to reduce the number of animals used and minimise animal suffering.

For this study, we used a mutant mouse line expressing Cre recombinase under the control of the Hdc promoter [B6.FVB(Cg)-Tg(Hdc-Cre)^IM1Gsat/Mmucd^ (RRID: MMRRC_037409-UCD)] (Hdc-Cre)^6^ as previous studies reported target specificity of 94.7% (albeit with penetrance of only 48.8%)^6^. Only male heterozygous Hdc-Cre mice were used for these experiments. All mice were group-housed in a temperature-controlled (23 °C ± 1 °C) and humidity-controlled (55.0% ± 5.0%) animal room under a 12:12 h light:dark cycle [lights on at 8:00 (ZT0), lights off at 20:00 (ZT12)] with ad libitum access to standard rodent chow (CLEA Japan, Tokyo, Japan) and water.

### Genotyping

Genotyping was performed in accordance with protocol MMRRC_032079-UCD. Briefly, genomic DNA was extracted from tail biopsies and analysed by polymerase chain reaction (PCR) using the KOD FX Neo enzyme (TOYOBO, Osaka, Japan). The PCR reaction protocol consisted of incubation at 94 °C for 2 min, followed by 35 cycles of 98 °C for 10 s, 60 °C for 30 s and 68 °C for 40 s under control of a T100 thermal cycler (Bio-Rad, Hercules, CA). The primer sequences were as follows: Hdc-Cre (575 bp): forward, 5′-AGC CTC CTC TGT CTG TCT GC; reverse, 5′-CCC CAG AAA TGC CAG ATT ACG TAT. WT (220 bp): forward, 5′-CTC ACC CAA ATC CAA CGA CT; reverse, 5′-GCA GTT GCC TAG CAA CAC AC.

### Surgery

Adult male Hdc-Cre mice (10‒14 weeks old) were anaesthetised using a mixture of 0.3 mg/kg medetomidine (Zenoaq, Fukushima, Japan), 4 mg/kg midazolam (Astellas, Tokyo, Japan) and 5 mg/kg butorphanol (Meiji Seika Pharma, Tokyo, Japan), and fixed on a digital stereotactic instrument (940-XYZ, David Kopf, Tujunga, CA). A 100-nL volume of vector solution was microinjected into each TMN (AP − 2.4 mm, RL ± 1.0 mm, DV − 5.1 mm from bregma). The vector solution contained either AAV8-hSyn-DIO-hM3Dq-mCherry (titre: 2.2 × 10^13^ vg/mL, 44361-AAV8; Addgene, Watertown, MA) (AAV-hM3Dq) or AAV8-DIO-hSyn-hM4Di-mCherry (titre: 2.5 × 10^13^ vg/mL, 4362-AAV8; Addgene) (AAV-hM4Di) to establish mouse models for chemogenetic activation and inhibition, respectively. Three weeks after microinjection, open field and resident–intruder tests were performed as described (“Behavioural experiments”). After the resident–intruder test, all mice were implanted with a head mount and electrodes (E363/76//NS/SPC, P1 Technologies, Manhattan Beach, CA) for EEG and electromyogram (EMG) recordings^55^. The EEG electrodes were inserted by threading through stainless steel screws while EMG electrodes were inserted directly into the neck extensor muscles.

### Histology

After completion of behavioural studies, mice were deeply anaesthetised with a medetomidine/midazolam/butorphanol mixture and transcardially perfused with 15 mL phosphate buffered saline (PBS) followed by 50 mL of 10% formalin in PBS (Wako, Osaka, Japan). Mouse brains were harvested immediately and incubated in 10% formalin/PBS overnight at 4 °C, followed by incubation in 10% formalin/PBS with 20% sucrose at 4 °C for 2 days. Brains were sliced into 3 series of 40-μm coronal sections using a freezing cryostat (CM3050, LEICA, Wetzlar, Germany) and processed for immunohistochemistry.

For immunohistochemistry using diaminobenzidine (DAB) as the chromogen, sections were incubated with c-Fos antibody (1:10,000; SC-52; Santa Cruz Biotechnology, Dallas, TX) for two nights or ds-Red antibody (1:10,000; Z2496N; Clontech Laboratory, Mountain View, CA) overnight at room temperature, followed by incubation in the appropriate biotin-SP-conjugated secondary antibody (1:1000; Jackson ImmunoResearch, West Grove, PA) for 1 h at room temperature. Sections were incubated for 75 min in avidin–biotin-complex reagent (1:1000; Vectastain ABC kit, Vector Laboratories, Burlingame, CA) at room temperature, washed and incubated in a solution of 0.06% DAB (Wako) and 0.02% H_2_O_2_ (Wako) for 2‒5 min. Then, PBS containing 0.05% CoCl_2_ (Wako) and 0.01% NiSO_4_ (NH_4_) (Wako) was added to the DAB solution for staining (black).

Sections were mounted on Superfrost glass slides (Matsunami, Osaka, Japan), dehydrated, cleared and cover-slipped using Multi Mount 480 (Matsunami). The mounted brain sections were scanned using a BZ-X700 microscope (Keyence, Osaka, Japan) and composite images were generated by BZ-X Analyzer software (Keyence).

### Behavioural experiments

All behavioural tests were performed with a two-arm crossover design by experimenters blinded to treatment history. Each microinjected mouse was examined first in the open-field test, followed by the resident–intruder test and sleep–wake cycle monitoring by EEG/EMG. Mice were randomly assigned to receive CNO or SA at the beginning of the first trial, then switched to the other drug for the second trial. Again, tests were analysed by experimenters blinded to mouse treatment history.

#### Open-field test

The open-field test was performed as previously described^34^. Briefly, mice were injected with CNO (0.3 mg/kg; Sigma, St Louis, MO) or saline vehicle (SA) as a control 30 min before the trial during the light period. Each mouse was placed into the centre of a 60 cm × 60 cm square arena and allowed to explore freely for 30 min. Total movement distance, average speed, total movement time and time spent in the central area were recorded and quantified using a video tracking system (EthoVision^®^ XT, Ver. 13, Noldus, Wageningen, Netherlands) by an experimenter blinded to treatment history. The time interval between first and second trials was 24 h which is long enough to wash out intraperitonially administered CNO^56^.

#### Resident–intruder test

The resident–intruder test was performed as previously described^8^. Briefly, resident mice were housed individually for 1 week before the testing day to increase territorial motivation. Then, the mouse was injected with CNO (0.3 mg/kg; Sigma) or SA 30 min before the trial during the light period. An unfamiliar male (intruder, C57BL6 WT, matched for age and weight) was introduced into the resident cage and the two mice were allowed to interact freely for 10 min. Aggression of the resident mouse was measured by the frequencies of ‘mounting’ (attempts to mount the intruder), ‘chasing’ and ‘biting’ (bites to dorsal/ventral regions of the intruder). The latency to first aggressive behaviour, number of aggressive behaviours and total duration of aggressive behaviours were measured. The resident mouse was transferred to a new clean cage immediately after the first trial and isolated to regain territorial motivation. The second trial was performed after 1 week of isolation. Another unfamiliar male mouse was used as an intruder in the second trial. All experimental data were video-recorded and analysed by an examiner blinded to treatment history.

#### Sleep recordings and analysis

A week after EEG/EMG electrode implantation, mice were connected to recording cables and habituated for 3 days in a cylinder chamber (22 cm in diameter) placed within a soundproof electrically shielded box (SP-BOX/S, Shinfactory, Fukuoka, Japan) maintained at constant temperature (23 °C ± 1 °C) and humidity (55.0% ± 5.0%) under a 12:12 h light–dark cycle (ZT0: 8:00, ZT12: 20:00) with food and water available ad libitum. The mice were injected with CNO (0.3 mg/kg; Sigma) or SA at 10:50 AM (10 min before ZT3) or 7:50 PM (10 min before ZT12), followed by EEG/EMG recording for 24 h. Following an interval of at least 3 days, mice were switched to the other drug intervention group for another 24 h of EEG/EMG recording. The EEG/EMG signals were amplified (Biotex, Kyoto, Japan), digitised (AD16-16U/PIC/EV, CONTEC, Osaka, Japan) and recorded by a Vital Recorder system (Kissei Comtec, Nagano, Japan). Only the first 6 h of the 24 h period were analysed. Briefly, collected data were divided into 12-s epochs and scored manually for one of three sleep or wake states, wake, NREM sleep, or REM sleep, using SleepSign 3 (Kissei Comtec). Time spent in each state, episode frequency and episode duration were calculated in 1-h bins. Latencies to the first NREM and REM sleep episodes were measured as the period following wakefulness evoked by intraperitoneal (i.p.) injection. To assess the differences in bout durations between CNO and SA group, we analysed the combined data of each vigilance state with cumulative probability distribution^57–59^. The frequencies of state transitions (Wake to NREM, NREM to Wake, NREM to REM and REM to Wake) were counted. The power spectral density of EEG signals in each stage was calculated with 0.25 Hz resolution by Fast Fourier Transform using SleepSign 3 software, then normalised for each animal by calculating the % power of each bin relative to the total power from 0 to 20 Hz.

### Statistical analysis

All statistical analyses were performed using GraphPad Prism^®^ version 8 (GraphPad Software, La Jolla, CA). In behavioural experiments, the CNO group was compared to the SA group by two-way repeated measures ANOVA followed by Sidak’s post hoc test or by Mann–Whitney U test as indicated in the figure legends. The distributions of bout duration in each vigilance state between CNO and SA groups were compared by Kolmogorov–Smirnov test. All data are presented as the mean ± standard error of the mean (S.E.M.) unless otherwise noted. Differences were considered significant at *P* < 0.05. All statistical analyses were performed by experiments blinded to animal treatment history.

## Supplementary Information

Supplementary Information.

## Data Availability

The datasets generated during and/or analysed during the current study are available from the corresponding author on reasonable request.

## Abbreviations

AAV: Adeno-associated virus
Arch: Archaerhodopsin
BNST: Bed nucleus of the stria terminalis
CeA: Central amygdala
CNO: Clozapine N-oxide
DAB: Diaminobenzidine
DREADDs: Designer receptor activated by designer drugs
EEG: Electroencephalogram
EMG: Electromyogram
HA: Histamine
Hdc: Histidine decarboxylase
Hnmt: Histamine *N*-methyltransferase
LH: Lateral hypothalamus
NREM: Non-rapid eye movement
OF: Open field
REM: Rapid eye movement
SCN: Suprachiasmatic nucleus
SI: Substantia innominate
SPZ: Subparaventricular zone
TMN: Tuberomamillary nucleus
VMHvl: Ventromedial hypothalamus, ventrolateral subdivision
vlPAG: Ventrolateral periaqueductal grey
ZT: Zeitgeber time
