# Supplementary material for: Chemogenetic modulation of histaminergic neurons in the tuberomamillary nucleus alters territorial aggression and wakefulness
Source: Sci Rep. 2021 Sep 9;11:17935. doi: 10.1038/s41598-021-95497-3 (PMC8429727; doi:10.1038/s41598-021-95497-3)
Supplement: Supplementary file 1 — Supplementary Information. [file 41598_2021_95497_MOESM1_ESM.docx]

**Supplementary informationss**

Supplementary figure 1


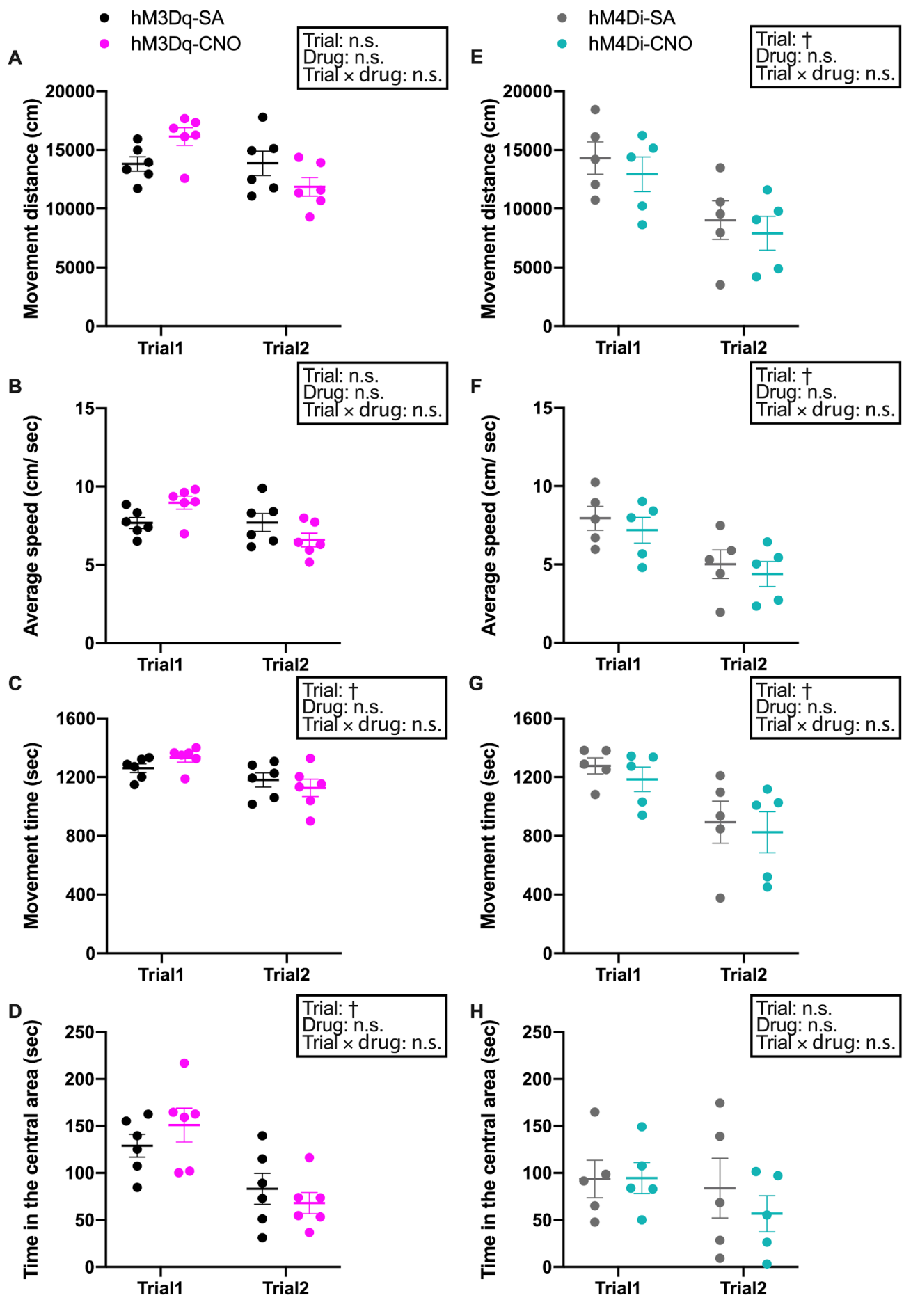


**Fig. S1 Chemogenetic activation of HA^TMN^ neurons enhanced locomotor activity in a novel open field but reduced locomotor activity in a pre-explored (non-novel) open field.**

Locomotor parameters were compared among Hdc-hM3Dq mice (A‒D, n = 6) and Hdc-hM4Di mice (E‒H, n = 5) for 30 min after CNO or SA injection by two-way RM ANOVA with main factors trial number (1 or 2) and drug treatment (CNO or SA), followed by Sidak’s *post hoc* tests. **A,** Total movement distance of Hdc-hM3Dq mice after CNO or SA injection (trial: *F* = 4.43, *P* = 0.062; drug: *F* = 0.087, *P* = 0.77; trial × drug interaction: *F* = 4.64, *P* = 0.057). **B**, Average locomotion speed of Hdc-hM3Dq mice after CNO or SA injection (trial: *F* = 4.43, *P* = 0.062; drug: *F* = 0.088, *P* = 0.77; interaction: *F* = 4.64, *P* = 0.057). **C**, Total movement time of Hdc-hM3Dq mice after CNO or SA injection (trial: *F* = 9.61, *P* = 0.01; drug: *F* = 0.046, *P* = 0.83; interaction: *F* = 1.88, *P* = 0.20). **D,** Total time spent in the central area by Hdc-hM3Dq mice after CNO or SA injection (trial: *F* = 21.58, *P* = 0.0009; drug: *F* = 0.046, *P* = 0.83; interaction: *F* = 1.78, *P* = 0.21). **E,** Total movement distance of Hdc-hM4Di mice after CNO or SA injection (trial: *F* = 9.44, *P* = 0.015; drug: *F* = 0.96, *P* = 0.36; interaction: *F* = 0.0062, *P* = 0.94). **F**, Average speed of Hdc-hM4Di mice after CNO or SA injection (trial: *F* = 9.44, *P* = 0.015; drug: *F* = 0.96, *P* = 0.36; interaction: *F* = 0.0062, *P* = 0.94). **G**, Total movement time of Hdc-hM4Di mice after CNO or SA injection (trial: *F* = 8.82, *P* = 0.018; drug: *F* = 0.68, *P* = 0.43; interaction: *F* = 0.0086, *P* = 0.93). **H,** Total time spent in the central area by Hdc-hM4Di mice after CNO or SA injection (trial: *F* = 1.8, *P* = 0.22; drug: *F* = 0.24, *P* = 0.64; interaction: *F* = 0.63, *P* = 0.45). Data are presented as mean ± S.E.M. (horizontal bars and error bars) with superimposed individual data points (closed circles). † *P* < 0.05 by two-way RM ANOVA with main factors ‘trial’ and ‘drug’.

Supplementary figure 2


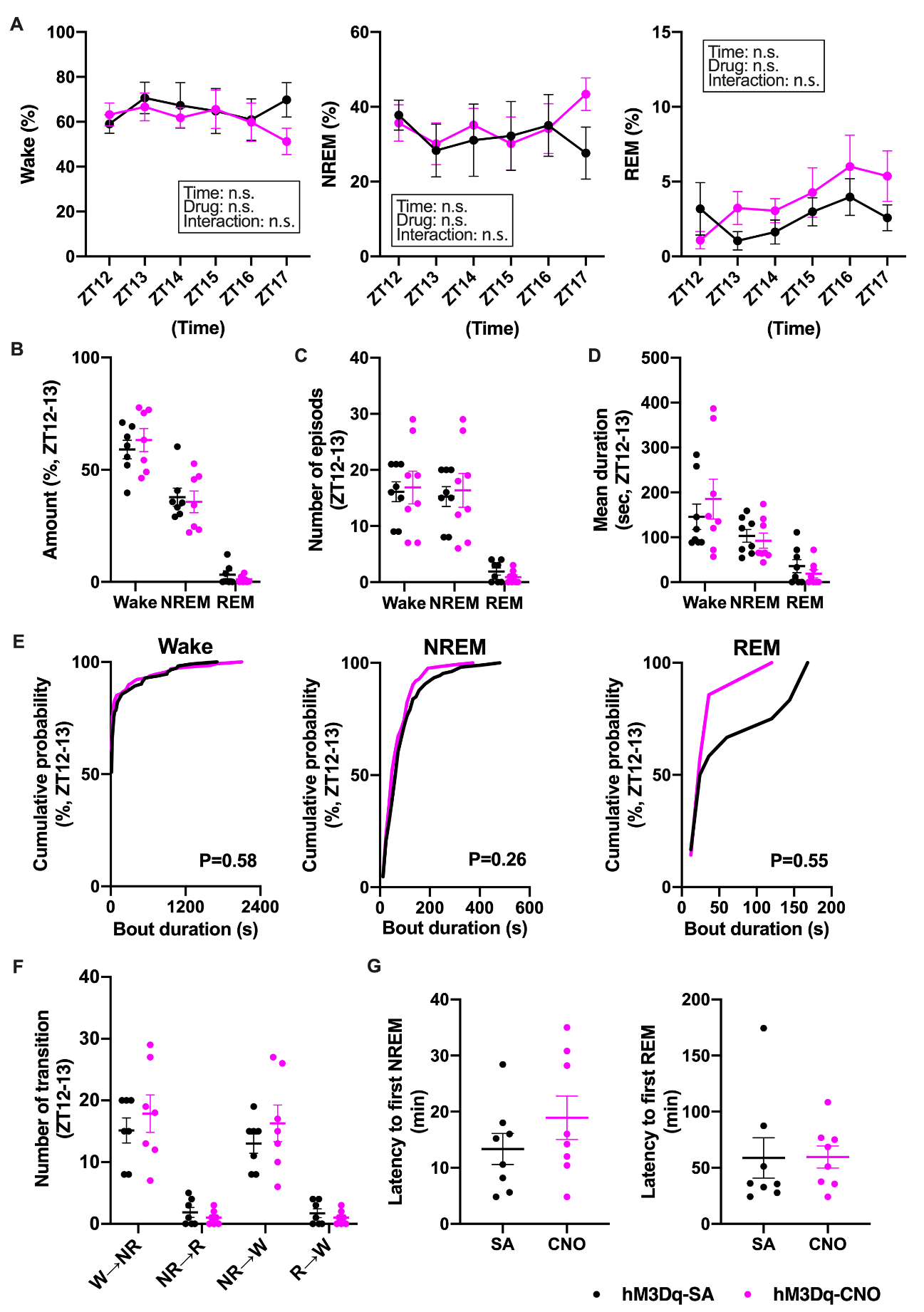


**Fig. S2 Chemoactivation of HA^TMN^ neurons in Hdc-hM3Dq** **mice (n = 7)** **did not alter the sleep–wake cycle during dark periods.**

All sleep parameters were compared by two-way RM ANOVA followed by Sidak's *post hoc* test except latencies and transitions, which were compared by Mann–Whitney U test (excerpt E). **A,** Hourly mean wake, NREM sleep and REM sleep times (% of total) after CNO or SA injection at ZT12 (wake **–** time: *F* = 0.52, *P* = 0.76; drug: *F* = 0.37, *P* = 0.55; interaction: *F* = 0.75, *P* = 0.59; NREM **–** time: *F* = 0.49, *P* = 0.78; interaction: *F* = 0.72, *P* = 0.61; drug: *F* = 0.20, *P* = 0.66; REM **–** time *F* = 2.02, *P* = 0.088; drug: *F* = 1.70, *P* = 0.22; interaction: *F* = 1.09, *P* = 0.37). **B,** Mean wake, NREM sleep and REM sleep times during ZT12–13 after CNO or SA injection at ZT12 (no significant difference). **C,** Number of wake, NREM sleep and REM sleep episodes during ZT12–13 after CNO or SA injection at ZT12. **D,** Mean wake, NREM sleep and REM sleep times during ZT12–13 after CNO or SA injection at ZT12. **E**, Vigilance state bout duration after CNO or SA injection at ZT12. Cumulative probability plots depict the bout durations’ relative distribution in each state during ZT12-13 (Wake *P* = 0.58, NREM *P* = 0.097 and REM *P* = 0.33 by Kolmogorov-Smirnov test). **F**, Sleep–wake state transitions after CNO or SA injection at ZT12 (wake ⭢ NREM *P* = 0.88, NREM ⭢ REM *P* = 0.60, NREM ⭢ wake *P* = 0.55, REM ⭢ wake *P* = 0.62). **G,** Latency to first NREM and REM sleep episodes during ZT12–13 after CNO or SA injection at ZT12 (no significant difference). All data expressed as mean ± S.E (except E).

Supplementary figure 3


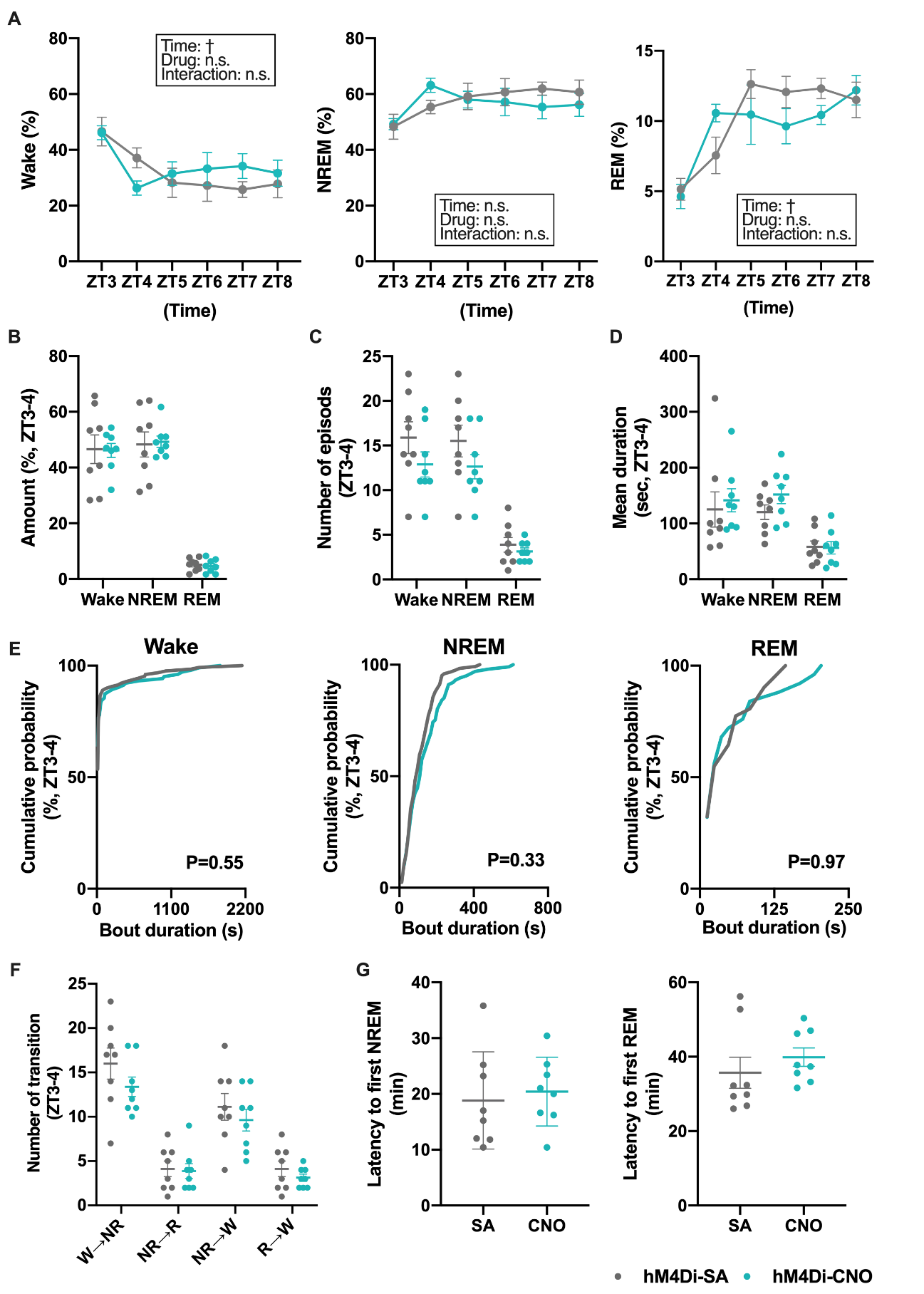


**Fig. S3 Chemogenetic inhibition of HA^TMN^ neurons in Hdc-hM4Di** **mice** **(n = 8)** **did not alter the sleep–wake cycle during light periods.**

All sleep parameters were compared by two-way RM ANOVA followed by Sidak's *post hoc* test except latencies and transitions, which were compared by Mann–Whitney U test (excerpt E). **A,** Hourly mean wake, NREM sleep and REM sleep times (% total) after CNO or SA injection at ZT3 (wake **–** time: *F* = 4.03, *P* = 0.0028; drug: *F* = 0.85, *P* = 0.37; interaction: *F* = 1.08, *P* = 0.38; NREM **–** time: *F* = 2.19, *P* = 0.065; drug: *F* = 0.36, *P* = 0.56; interaction: *F* = 0.86, *P* = 0.51; REM **–** time: *F* = 10.29, *P* < 0.0001; drug: *F* = 0.99, *P* = 0.34; interaction: *F* = 1.63, *P* = 0.16). **B,** Mean wake, NREM sleep and REM sleep times (% total) during ZT3–4 after CNO or SA injection at ZT3. **C,** Number of wake, NREM sleep and REM sleep episodes during ZT3–4 after CNO or SA injection at ZT3. **D,** Mean wake, NREM sleep and REM sleep durations (%total) during ZT3–4 after CNO or SA injection at ZT3. **E**, Vigilance state bout duration after CNO or SA injection at ZT3. Cumulative probability plots depict the bout durations’ relative distribution in each state during ZT3-4 (Wake *P* = 0.58, NREM *P* = 0.26 and REM *P* = 0.55 by Kolmogorov-Smirnov test. **F**, Sleep–wake state transitions after CNO or SA injection at ZT3 (wake ⭢ NREM *P* = 0.30, NREM ⭢ REM *P* = 0.79, NREM ⭢ wake *P* = 0.47, REM ⭢ wake *P* = 0.95). **G,** Latency to first NREM and REM sleep episodes during ZT3–4 after CNO or SA injection at ZT3 (no significant difference). All data expressed as mean ± S.E (excerpt E). † *P* < 0.05 by two-way RM ANOVA for ‘time’ and ‘drug’.

Supplementary figure 4


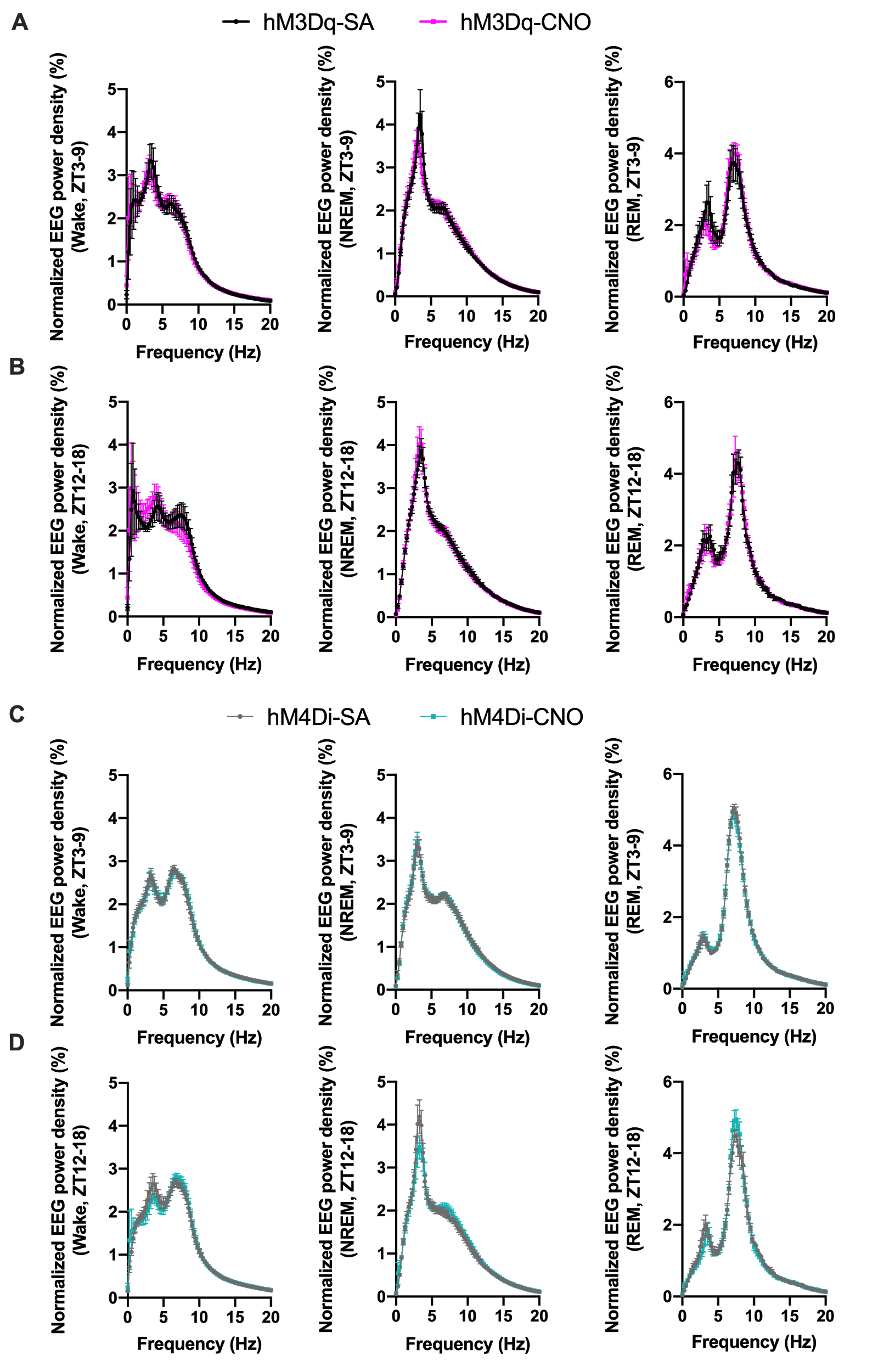


**Fig. S4 Power spectra of wake and each sleep stage during dark and light periods after CNO administration to Hdc-hM3Dq and Hdc-hM4Di mice.**

**A,** Power spectra of wake, NREM sleep and REM sleep periods for Hdc-hM3Dq mice during ZT3-9 after CNO or SA injection at ZT3 (n = 6; wake *P* = 0.85, NREM *P* = 0.85, REM *P* > 0.9999 all by Mann–Whitney U test). **B,** Power spectra of wake, NREM sleep and REM sleep periods for Hdc-hM3Dq mice during ZT12–18 after CNO or SA injection at ZT12 (n = 6; wake *P* = 0.99, NREM *P* = 0.91, REM *P* = 0.92, all by Mann–Whitney test). **C,** Power spectra of wake, NREM sleep and REM sleep periods for Hdc-hM4Di mice during ZT3–9 after CNO or SA injection at ZT3 (n = 7; wake *P* = 0.97, NREM *P* = 0.99, REM *P* = 0.93, all by Mann–Whitney U test). **D,** Power spectra of wake, NREM sleep and REM sleep periods for Hdc-hM4Di mice during ZT12–18 after CNO or SA injection at ZT12 (n = 7; wake *P* = 0.96, NREM *P* = 0.99, REM *P* = 0.78, all by Mann–Whitney U test).

Supplementary table 1

**Table S1 Hourly mean number and duration of wake, NREM sleep and REM sleep episodes in Hdc-hM3Dq mice during the dark and light periods after CNO or SA injection**


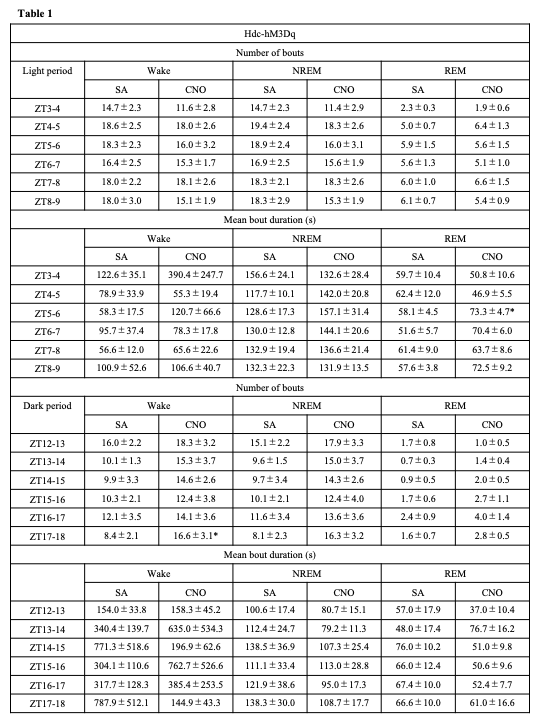


CNO or SA was administered i.p. at ZT3 for the light period test and at ZT12 for the dark period test.

Supplementary table 2

**Table S2 Hourly mean number and duration of wake, NREM sleep and REM sleep episodes in Hdc-hM4Di mice during the dark and light periods after CNO or SA injection**


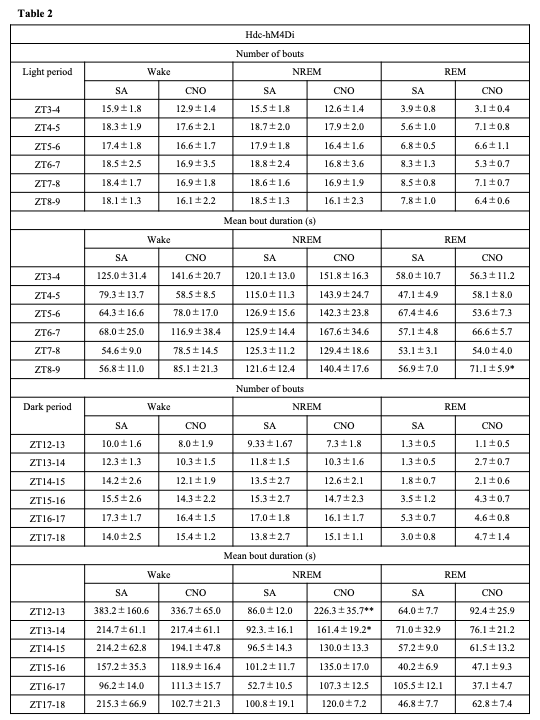


CNO or SA was administered intraperitoneally at ZT3 for the light period test and at ZT12 for the dark period test. Data presented as mean ± S.E.M. **P* < 0.05, ** *P* < 0.01 by Mann–Whitney U test.
